# Cotton straw biochar and compound *Bacillus* biofertilizer reduce Cd stress on cotton root growth by regulating root exudates and antioxidant enzymes system

**DOI:** 10.3389/fpls.2022.1051935

**Published:** 2022-11-15

**Authors:** Yongqi Zhu, Xin Lv, Tiansheng Li, Mingtao Zhong, Jianghui Song, Haijiang Wang, Jing Cui

**Affiliations:** College of Agriculture, Shihezi University, Shihezi, Xinjiang, China

**Keywords:** antioxidant enzyme activities, cadmium stress, metabolites, metabolomics, root exudates

## Abstract

**Introduction:**

Cotton straw biochar (biochar) and compound *Bacillus* biofertilizer (biofertilizer) have attracted wide attentions in the remediation of heavy metal-contaminated soils in recent years. However, few studies have explored the metabolomics of lateral roots of Cd-stressed cotton to determine the mechanism of biochar and biofertilizer alleviating Cd stress.

**Methods:**

In this pot experiment, biochar and biofertilizer were applied to the soils with different Cd contamination levels (1, 2, and 4 mg kg^-1^). Then, the responses of cotton root morphology, vitality, Cd content, and antioxidant enzyme activities were analyzed, and the mechanism of biochar and biofertilizer alleviating Cd stress was determined by metabolomic analysis.

**Results:**

The results showed that exogenous Cd addition decreased the SOD and POD activities in cotton taproot and lateral root. Besides, with the increase of soil Cd content, the maximum Cd content in taproot (0.0250 mg kg^-1^) and lateral root (0.0288 mg kg^-1^) increased by 89.11% and 33.95%, respectively compared with those in the control (*p<* 0.05). After the application of biochar and biofertilizer, the SOD and POD activities in cotton taproot and lateral root increased. The Cd content of cotton taproot in biochar and biofertilizer treatments decreased by 16.36% and 19.73%, respectively, and that of lateral root decreased by 13.99% and 16.68%, respectively. The metabolomic analysis results showed that the application of biochar and biofertilizer could improve the resistance of cotton root to Cd stress through regulating the pathways of ABC transporters and phenylalanine metabolism.

**Discussion:**

Therefore, the application of biochar and biofertilizer could improve cotton resistance to Cd stress by increasing antioxidant enzyme activities, regulating root metabolites (phenols and amino acids), and reducing Cd content, thus promoting cotton root growth.

## Introduction

Heavy metals, especially Cd, can interfere with plant physiological processes by stimulating H_2_O_2_ generation and inhibit plant growth (Lai et al., 2020). Previous studies have shown that soil Cd pollution in farmlands is closely related to the excessive application of chemical fertilizers and pesticides and wastewater irrigation (Hong et al., 2020). Cd in soil is easily absorbed by plant roots and transmitted upward to other tissues. High content of Cd can inhibit plant seed germination and root growth, and lead to leaf withering, lipid peroxidation, and reduced enzyme activity (Rizwan et al., 2016; Berthet et al., 2018). Cd enriched in crops, ultimately, may result in the decline of yield and quality, and endanger human health through the food chain.

Plant root, the organ for fixation, absorption, and metabolism of exogenous elements, plays an important role in the physical, chemical, and biological processes that occur in the rhizosphere. Besides, it also actively responds to soil environment changes (Kopittke and Wang, 2017). Song et al. (2017) reported that the absorption of Cd by plant roots led to an increase in malondialdehyde (MDA) content and a decrease in antioxidant enzyme activity in plant roots through increasing the synthesis of protein and damaging nucleic acid and lipid cell membrane. Arif et al. (2019) found that major organic compounds involved in the chelation of metal ions, such as phytochelatins (PCs), metallothioneins (MTs), organic acids, amino acids, and cell wall proteins/pectins/polyphenols, could reduce the concentrations of metal ions, thereby reducing heavy metal-induced phytotoxicity. In addition, heavy metals can induce plant roots to produce secondary metabolites. Some studies have shown that the contents of alanine, proline, serine, putrescine, and phytohormones (campesterol, β- sitosterol, isoflavones, and α- tocopherol) increase significantly under Cd stress (Arif et al., 2019; Zhou et al., 2019).

Biochar, a carbon-rich organic material, can stimulate the growth of plant roots. Previous studies have shown that the application of biochar could increase the biomass, length, tip number, and density of plant roots, and reduce the available Cd in soil by 97.31% (Yang et al., 2016; Farrar et al., 2021). Biofertilizer, containing living cells or latent cells (Nascimento et al., 2020), could transform bioavailable heavy metals in the soil into unavailable states through microbial adsorption and fixation. For example, Iftikhar et al. (2019) reported that after the application of biofertilizer, the available Cd content in soil decreased by 75.59% compared with that in the control. Zhu et al. (2022) reported that the Cd content in cotton roots and leaves decreased significantly after biofertilizer application.

According to the statistics of the International Cotton Advisory Committee (ICAC), China’s cotton output in 2020 was 5.9237 million tons, accounting for 23.43% of global cotton output. Xinjiang is the main cotton producing area of China. At present, the contents of Pb, Zn, Cd, and Cu are very high in cotton fields in Northern Xinjiang, China, and the Cd content exceeds the soil background value in 20.38% of the farmland soils. Chen et al. (2019) reported that the soil Cd, Hg, Cr, and Ni contents in Minfeng County, Xinjiang, China were 1.147, 1.124, 1.116, and 1.041 times of the background values of the soil in Xinjiang, respectively. Therefore, the ecological risk of soil heavy metal pollution is very high in Xinjiang, China (Liu et al., 2020).

Many studies have used biochar and biofertilizer as modifiers in the remediation of heavy metal-contaminated soils, and explored changes in soil physical and chemical properties and heavy metal bioavailability (Sun et al., 2017; Elsayed et al., 2020). However, there are few studies on the effects of biochar and biofertilizer on the morphological and physiological characteristics of cotton roots as well as cotton root-secreted metabolites under Cd stress. Therefore, in this study, the effects of exogenous Cd, biochar, and biofertilizer on the morphology, vitality, and antioxidant enzyme activities of cotton roots were determined, and the mechanism of biochar and biofertilizer alleviating Cd stress was explored by metabolomics analysis. We hypothesized that: (1) the application of biochar and biofertilizer might reduce the absorption of Cd in soil by cotton roots, and (2) biochar and biofertilizer might alleviate Cd stress by regulating the secretion of metabolites by cotton roots. This study will provide guidance for the remediation of Cd-contaminated soils in arid regions.

## Materials and methods

### Experimental site

The pot experiment was conducted at the Experimental Station of Shihezi University in Xinjiang, China (44°18′42.37″N, 86°03′20.72″E). This region has a temperate continental climate, with an annual average temperature of 7.5 ~ 8.2°C, sunshine duration of 2318 ~ 2732 h, frost free period of 147 ~ 191 d, annual rainfall of 180 ~ 270 mm, and annual evaporation of 1000 ~ 1500 mm. The main crops were cotton, wheat, corn, and sugar beet.

### Preparation of experimental materials

Soil was collected from a cotton field continuously cropped for 25 years in the study area. The texture was clay loam. The physical and chemical properties are shown in **Table 1**. Then, exogenous CdCl_2_·2.5 H_2_O (2.44 g, analytical purity) was dissolved in distilled water, shaken well, and diluted into 1,000 mL to obtain 1.2 g·L^-1^ of Cd^2+^ solution. After that, 10 mL, 20 mL, and 40 mL of the solution were separately mixed with 12 kg collected soil to prepare the test soils with different Cd contents (0.25 (H0), 1 (H1), 2 (H2), and 4 (H3) mg kg^-1^). The H1, H2 and H3 levels are 3, 6, and 11 times the global average soil Cd content (Adriano, 2001; Rawlins et al., 2012). The prepared soils were used for the experiment after 60 days.

**Table 1:** Physicochemical properties of modifiers and soil.

Property	Cotton-straw biochar	Compound *Bacillus* biofertilizer	Soil
pH	9.50	7.8	7.76
Total nitrogen (g·kg^-1^)	0.89	91	0.46
Total P (g·kg^-1^)	2.54	62.2	0.82
Organic matter (g·kg^-1^)	625	422	14.73
Total K (g·kg^-1^)	8.62	86.1	246.83
Total Cd (mg·kg^-1^)	0.002	0.0001	0.25
Available Cd	–	–	0.121
Total salinity (g·kg^-1^)	–	–	3.36
Carboxyl (mmol·g^-1^)	0.20	–	–
Lactone (mmol·g^-1^)	0.25	–	–
Phenolic hydroxyl (mmol·g^-1^)	0.21	–	–
The Colony-Forming Units	–	>20 billion·g^-1^	–

The biochar (B) was prepared by using cotton straw according to the method of Lehmann et al. (2011). Biochar was air-dried and passed through a 5 mm sieve before the determination of pH, organic matter, total nitrogen, total phosphorus, total potassium, and total Cd (Sun et al., 2007) (**Table 2**). Biofertilizer (J) was purchased from the Lvlong Biotechnology Co., Ltd, Shandong, China. The number of viable bacteria in J was greater than 20 billion·g^-1^, the dominant bacterial species was *Bacillus*, the proportion of miscellaneous bacteria was less than 0.4%, the water content was less than 10%, the pH was 7.8, the total Cd content was 0.0001 mg·L^-1^, the total nitrogen content was 900 mg·L^-1^, and the organic carbon content was 3791 mg·L^-1^ (Zhu et al., 2022).

**Table 2:** Experimental design.

Treatments	Cd (mg·kg^-1^)	Biochar (%)	Biofertilizer (%)
H0T (Control)	0.25	0	0
H0B	0.25	3%	0
H0J	0.25	0	1.5%
H1T	1	0	0
H1B	1	3%	0
H1J	1	0	1.5%
H2T	2	0	0
H2B	2	3%	0
H2J	2	0	1.5%
H3T	4	0	0
H3B	4	3%	0
H3J	4	0	1.5%

T, no modifiers; B, 3% biochar was applied; J, 1.5% biofertilizer was applied; H0, no Cd; H1, 1 mg·kg^-1^ of Cd was applied; H2, 2 mg·kg^-1^ of Cd was applied; H3, 4 mg·kg^-1^ of Cd was applied.

### Experimental design

This experiment was conducted from 2019 to 2020. There were twelve treatments totally (**Table 1**), and each treatment had five replicates. In April 25, 2019, 3% B (360 g/pot, w/w) and 1.5% J (180 g/pot, w/w) were mixed with the prepared Cd-contaminated soils respectively (Kim et al., 2015; Zhu et al., 2022). Then, the soils were transferred into pots with a height of 40 cm and a diameter of 25 cm. After one week, deionized water was used for irrigation to maintain soil water content at 60% of the field capacity. After 60 days, B and J were applied to the soils according to the experimental design, and compound fertilizer (555 kg hm^-2^; N-P_2_O_2_-K_2_O, 17-17-17), potassium polyacrylate (4.8 kg hm^-2^), and 50% of nitrogen fertilizer (urea, 345 kg hm^-2^) were also applied. On May 2, 2019, ten cotton seeds (variety Xinluzao 53) were sown in each pot. During the whole growth period, the soil water content was maintained at 60% of the field capacity. The rest nitrogen fertilizer was applied after planting. Cotton plants were harvested on October 1, 2019, and the soils in the pots were put outdoors for winter.

In 2020, cotton planting and the application of fertilizers, B, J, and field managements were consistent with those in 2019, but Cd was no more applied. All cotton root were sampled after 120 d cultivation (on September 10, 2020). Then, the roots were washed with water after removing impurities, put into ice bags, and brought back to the lab. Twelve taproot samples and twelve lateral root samples were obtained from each treatment. The total biomass of each root (RB) was weighed and recorded. After that, half of the taproot and one third of the lateral root were used for the determination of antioxidant enzyme activity and root vitality. The other half of the taproot and one third of the lateral root was first dried in an oven at 105°C for 2 h and then dried to constant weight at 85°C for the determination of Cd content. The remaining lateral roots were reserved at -80°C for metabolomics analysis. Following formulas were used for calculating root length density (RD, cm·cm^-3^), root tissue mass density (RTD, mg·cm^-3^), and specific root length (SRL, m•g^-1^) (Birouste et al., 2014).


 (1) $$\text{RD}=\frac{\text{Root length}}{\text{Soil sample volume}}$$

(2) $$\text{RTD}=\frac{\text{Root dry mass}}{\text{Root volume}}$$

(3) $$\text{SRL}=\frac{\text{Root length}}{\text{Root dry mass}}$$

### Determination methods

Accurately weighed 0.5 g dried root (taproot and lateral root) was digested with the mixture of nitric acid and perchloric acid (2:1, v/v) (Yang et al., 2016), and then the Hitachi Z2000 graphite atomic absorption spectrophotometer (Z2000, Hitachi, Tokyo, Japan) was used to determine the Cd content.

The standard curve of graphite atomic absorption spectrometry: Accurately absorb cadmium standard solution (Meryer (Shanghai) Chemical Technology Co., Ltd. Shanghai) (100 μg L^-1^) 0, 0.5, 1.0, 1.5, 2.0, 3.0 mL in 100 mL volumetric flask, the standard series solutions containing 0, 0.50, 1.0, 1.5, 2.0 and 3.0 μg L^-1^ cadmium were obtained by constant volume of 2% HNO_3_ to the scale. The calibration curves were linear in the range of 0~3.0μg/L for cadmium, R^2 =^ 0.9997, the determination of metals with limits of detection 0.23μg/L. The precision of the procedures was also calculated as 7.6%, the recovery percentages varied between 105% and 109%.

The activity of antioxidant enzymes in roots was determined by spectrophotometry. Superoxide dismutase (SOD) activity was determined using the method of Paoletti et al. (1986) based on NBT photochemical reduction. The catalase (CAT) and peroxidase (POD) activities were determined by using the method of Cakmak and Marschner (1992). The content of malondialdehyde (MDA) was determined by the content of thiobarbituric acid reactive substances (TBARS) according to the method of Bharwana et al. (2014). Electrolyte leakage rate (REC) was determined by the method of Bharwana et al. (2014).

The root vitality was determined by triphenyltetrazole chloride (TTC) method (Liu et al., 2008). Root samples were washed with distilled water and dried with thin paper. Then, root sample (0.5 g) was completely immersed in a solution composed of 5 mL of 0.4% TTC and 5 mL of phosphate buffer (0.06 mol·L^-1^, pH: 7.0), and placed in the dark at 37°C for 3 h. After that, 2 mL of 1 mol·L^-1^ sulfuric acid was added to stop the chemical reaction. The roots were removed, and the residue was homogenized in a mortar containing 4 mL of ethyl acetate. The red extract was transferred to test tube, and 10 mL of ethyl acetate was added, followed by spectrophotometric measurement at 485 nm. The amount of tetrazolium reduced in the standard reaction was quantified with absorbance to calculate the root vitality.

### Metabolonomic analysis

The test results showed the vitality and Cd content of cotton lateral roots were higher than those of taproot. Therefore, the metabonomics of cotton lateral roots was determined in this study.

The H0T, H4T, H4B, and H4J treatments were selected for metabolomic analysis. Small molecular metabolites including polar and non-polar metabolites were extracted from 1.0 g of soil using a mixture of methanol and H_2_O (v/v=3:1) and then a mixture of ethyl acetate and H_2_O (v/v=1:1). After that, all metabolites were dissolved in 30 μL of methoxylamine hydrochloride (20 m·mL^-1^ in pyridine) before an incubation at 80 °C for 30 min and an incubation with 40 μL of BSTFA (Bis(trimethylsilyl)trifluoroacetamide) reagent (1% TMCS (Trimethylchlorosilane), v/v) at 70 °C for 1.5 h. After cooling to room temperature, 5 μL of FAMEs (dissolved in chloroform) were added to the quality control sample. The composition and content of soil metabolites were analyzed in a gas chromatograph (GC) system (Agilent 7890, Santa Clara, USA) equipped with a DB-5 mass spectrometer (MS) capillary column (inner diameter: 30 m × 250 μm, film thickness: 0.25 μm) (J&W Scientific, Folsom, CA, USA) and linked with a Pegasus high-throughput time-of-flight mass spectrometer (Pegasus^®^ HT TOF-MS, LECO, MI, USA). Helium, as carrier gas, was accessed at a constant flow rate of 1.0 mL·min^-1^. Full-scan mode with a range of 50-500 m/z and a rate of 20 spectra·s^-1^ were used for MS data acquisition. Finally, following the Metabolomics Standards Initiative (MSI) convention, the extraction, alignment, identification, and integration of peaks were implemented on the LECO-Fiehn Rtx5 database (Zhu et al., 2022). The comparative analyses included H3T vs CK, H3J vs H3T, and H3B vs H3T.

### Data analysis

Significance of the differences in root biomass, specific root volume/area/weight, root vitality, root Cd, and antioxidant enzyme activities was determined using Duncan test at *p<* 0.05 (SPSS version 20.0 software, Inc., Chicago, IL, USA). All charts (bar chart, OPLS-DA chart, boxplot, metabolite expression chart, heatmap, and Venn diagram) were drawn using Origin 8.0 (Origin Lab, Massachusetts, USA). Image layout was completed using Adobe Illustrator CS6 (Adobe Systems Incorporated, USA).

## Results

### Root structure

Exogenous Cd addition reduced cotton RB and RTD, but had no obvious effect on RD and SRL. Cotton RB and RTD in the H3T treatment decreased by 10.46% and 21.81% (*p*< 0.05), respectively compared with those in the H0T treatment. The application of B and J increased cotton RB, RD, SRL, and RTD. Cotton RB, RD, SRL, and RTD in the H3B treatment increased by 5.23%, 15.25%, 14.29%, and 11.83% (*p*< 0.05), respectively compared with those in the H3T treatment, and RB, RD, SRL, and RTD in the H3J treatment increased by 9.25%, 16.39%, 15.69%, and 13.24% (*p*< 0.05), respectively compared with those in the H3T treatment. The results of regression analysis showed that Cd and modifier application had effects on cotton RB (*p*< 0.05), RD (*p<* 0.01), SRL (*p<* 0.01), and RTD (*p<* 0.01) (**Table 3**).

**Table 3:** Cotton root structure in different treatments.

Treatments	RB (g)	RD (cm·cm^-3^)	SRL (m•g^-1^)	RTD (mg·cm^-3^)
H0T	0.985±0.0493 bcd	124.699±6.235 cd	119.030±5.951 b	0.454±0.0227 d
H0B	1.012±0.0506 b	154.946±7.747 b	135.342±6.767 a	0.724±0.0362 b
H0J	1.094±0.0547 a	161.549±8.077 a	142.358±7.118 a	0.854±0.0427 a
H1T	0.934±0.0467 bcde	151.457±5.773 def	120.252±6.013 b	0.403±0.0201 e
H1B	0.967±0.0484 bcd	134.569±6.728 c	133.855±6.693 a	0.523±0.0262 c
H1J	1.001±0.0501 b	148.255±7.413 b	138.545±6.927 a	0.683±0.0342 b
H2T	0.902±0.0453 def	108.646±5.432 f	119.236±5.962 b	0.395±0.0198 e
H2B	0.905±0.0541 cdef	120.546±6.027 de	130.455±6.523 ab	0.487±0.0243 cd
H2J	0.992±0.0496 bc	121.456±6.072 de	140.745±7.037 a	0.499±0.0250 cd
H3T	0.822±0.0411 f	95.646±4.782 g	119.655±5.983 b	0.355±0.0177 e
H3B	0.865±0.0433 ef	110.235±5.512 ef	136.754±6.838 a	0.397±0.0199 e
H3J	0.898±0.0449 def	111.326±5.566 ef	138.425±6.921 a	0.402±0.0201 e
Regression Tests (significance)				
H	**	**	**	**
B	*	**	**	**
J	*	**	**	**
H*BJ	**	**	**	**

T, no modifiers; B, 3% biochar was applied; J, 1.5 % biofertilizer was applied; H0, no Cd; H1, 1 mg·kg^-1^ of Cd was applied; H2, 2 mg·kg^-1^ of Cd was applied; H3, 4 mg·kg^-1^ of Cd was applied. RB, root biomass; RD, root length density (cm·cm^-3^); RTD, root tissue mass density (mg·cm^-3^); SRL, specific root length (m•g^-1^). Different lowercase letters in the same column indicate significant differences. **, p< 0.01; *, 0.01< p< 0.05; ns, p ≥ 0.05. The same below.

### Vitality of cotton taproot and lateral root

**Figure 1:**
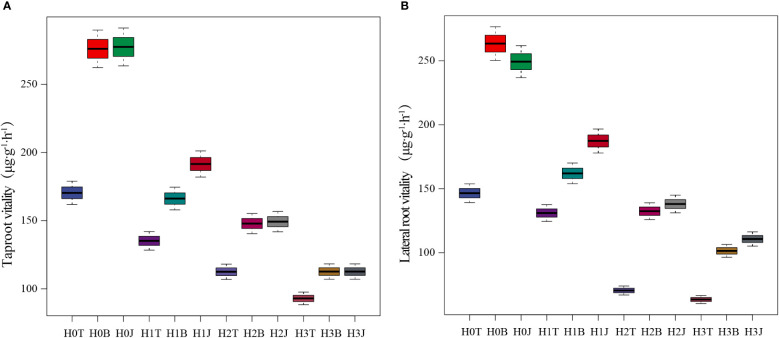
Effect of biochar and biofertilizer applications on the vitality of cotton taproot **(A)** and lateral root **(B)**. Notes: T, no modifiers; B, 3% biochar was applied; J, 1.5 % biofertilizer was applied; H0, no Cd; H1, 1 mg·kg-1 of Cd was applied; H2, 2 mg·kg-1 of Cd was applied; H3, 4 mg·kg-1 of Cd was applied. The same below.

The vitality of cotton taproot and lateral root were affected by exogenous Cd to varying degrees (**Figure 1**). The vitality of cotton taproot and lateral root in the H1T, H2T, and H3T treatments decreased compared with those in the H0T treatment (*p*< 0.05). With the increase of Cd content, the vitality of cotton taproot and lateral root showed a downward trend, and were the lowest in the H3T treatment (92.06 and 63.38 µg·g^-1^·h^-1^, respectively). However, the application of B and J increased the vitality of cotton taproot and lateral root (*p*< 0.05). The vitality of cotton taproot in the H3B and H3J treatments increased by 21.20% and 21.25% (*p*< 0.05), respectively, and that of lateral root increased by 60.01% and 74.63% (*p<* 0.05), respectively, compared with those in the H3T treatment.

### Oxidative stress response of cotton taproot and lateral root

**Figure 2:**
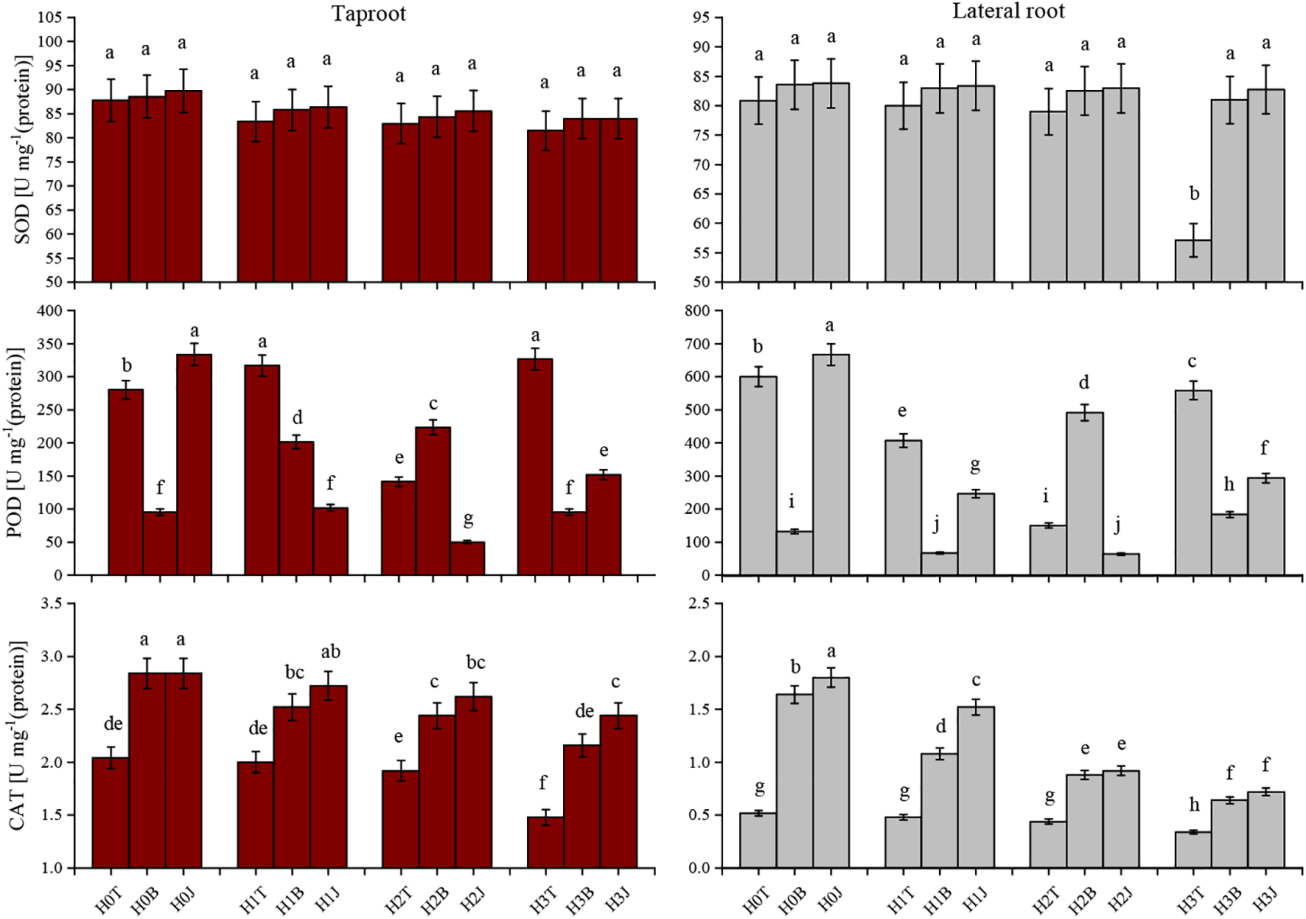
Effect of biochar and biofertilizer applications on cotton root antioxidant defense system. Values are means ± SE (n=5). Different lowercase letters indicate significant differences at P < 0.05 (the Duncan test).

In this study, exogenous Cd addition reduced the SOD activity and CAT activity of cotton taproot and lateral root. With the increase of Cd content, the SOD and CAT activities of cotton root showed a decreasing trend. The SOD activity of cotton taproot and lateral root in the H3T treatment decreased by 7.17% and 5.89% (*p* > 0.05), respectively, while the CAT activity decreased by 27.45% and 34.63%, respectively (*p<* 0.05) compared with those in the H0T treatment. The MDA content and REC increased with the increase of Cd content (*p<* 0.05), reaching the maximum in H3T treatment. The highest MDA content of cotton taproot and lateral root were 11.39 and 14.98 µmol·mg^-1^ (protein), respectively, and the highest REC were 16.67% and 15.03%, respectively (**Figure 2**).

**Figure 3:**
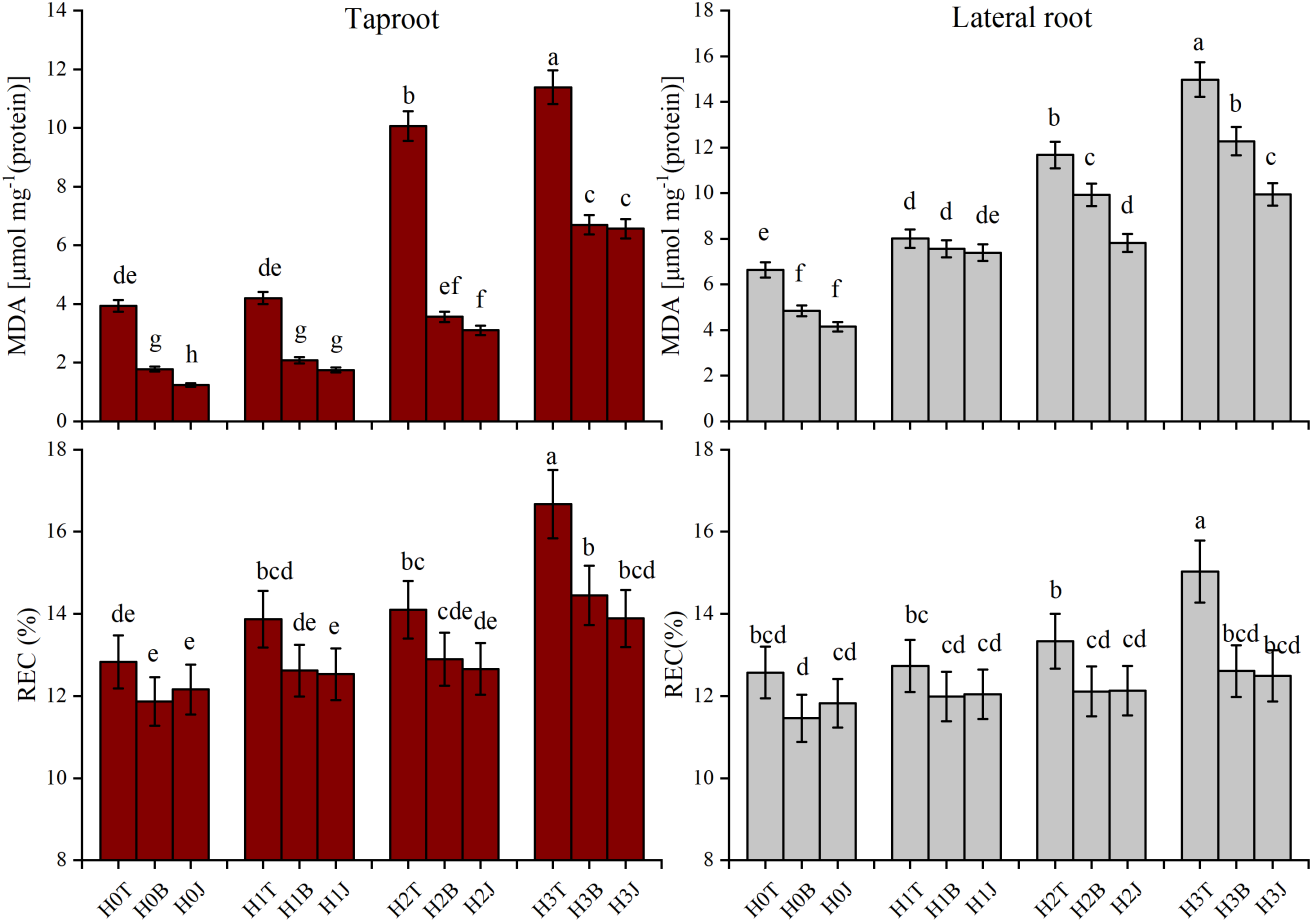
Effect of biochar and biofertilizer applications on cotton root malondialdehyde (MDA) and electrolyte leakage rate (REC). Values are means ± SE (n=5). Different lowercase letters indicate significant differences at P < 0.05 (the Duncan test).

The application of B and J increased the activities of SOD and CAT in taproot and lateral root. The activities of SOD and CAT of taproot in the H3B treatment increased by 3.07% (*p* > 0.05) and 45.95% (*p<* 0.05), respectively, and those of lateral root increased by 6.37% (*p* > 0.05) and 88.24% (*p<* 0.05), respectively compared with those in the H3T treatment. The activities of SOD and CAT of taproot in the H3J treatment increased by 3.072% (*p* > 0.05) and 64.86% (*p<* 0.05), respectively, and those of lateral root increased by 8.724% (*p* > 0.05) and 111.7% (*p<* 0.05), respectively compared with those in the H3T treatment (**Figure 2**). The MDA content of taproot and lateral root in the H3B treatment decreased by 41.12% and 18.02%, respectively (*p<* 0.05), and the REC of taproot and lateral root decreased by 13.32% and 16.11%, respectively (*p<* 0.05), compared with those in the H3T treatment. The MDA content of taproot and lateral root in the H3J treatment decreased by 42.32% and 33.58%, respectively (*p<* 0.05), and the REC of taproot and lateral root decreased by 16.68% and 16.91%, respectively (*p<* 0.05), compared with those in the H3T treatment. There was no difference in the MDA content and REC between other treatments (*p* > 0.05) (**Figure 3**).

### Changes in Cd content in taproot and lateral root of cotton after biochar and biofertilizer applications

Modifiers and exogenous Cd had obvious influences on the enrichment of Cd in cotton roots (**Figure 1**). The Cd contents of cotton taproot and lateral root in the H1T, H2T, and H3T treatments increased compared with those in the H0T treatment (*p*< 0.05). The Cd content of cotton taproot (0.0250 mg kg^-1^) and lateral root (0.0288 mg kg^-1^) in the H3T treatment were the highest, increasing by 89.11% and 33.95%, respectively (*p*< 0.05) compared with those in the H0T treatment. The application of B and J significantly reduced the accumulation of Cd in cotton roots (*p*< 0.05). The taproot Cd content in the H3B and H3J treatments reduced by 13.32% and 19.47%, respectively, and the lateral root Cd content reduced by 14.24% and 20.49%, respectively, compared with those in the H3T treatment (*p*< 0.05).

### Metabolic profiling of lateral roots exposed to biochar, biofertilizer, and Cd

**Figure 4:**
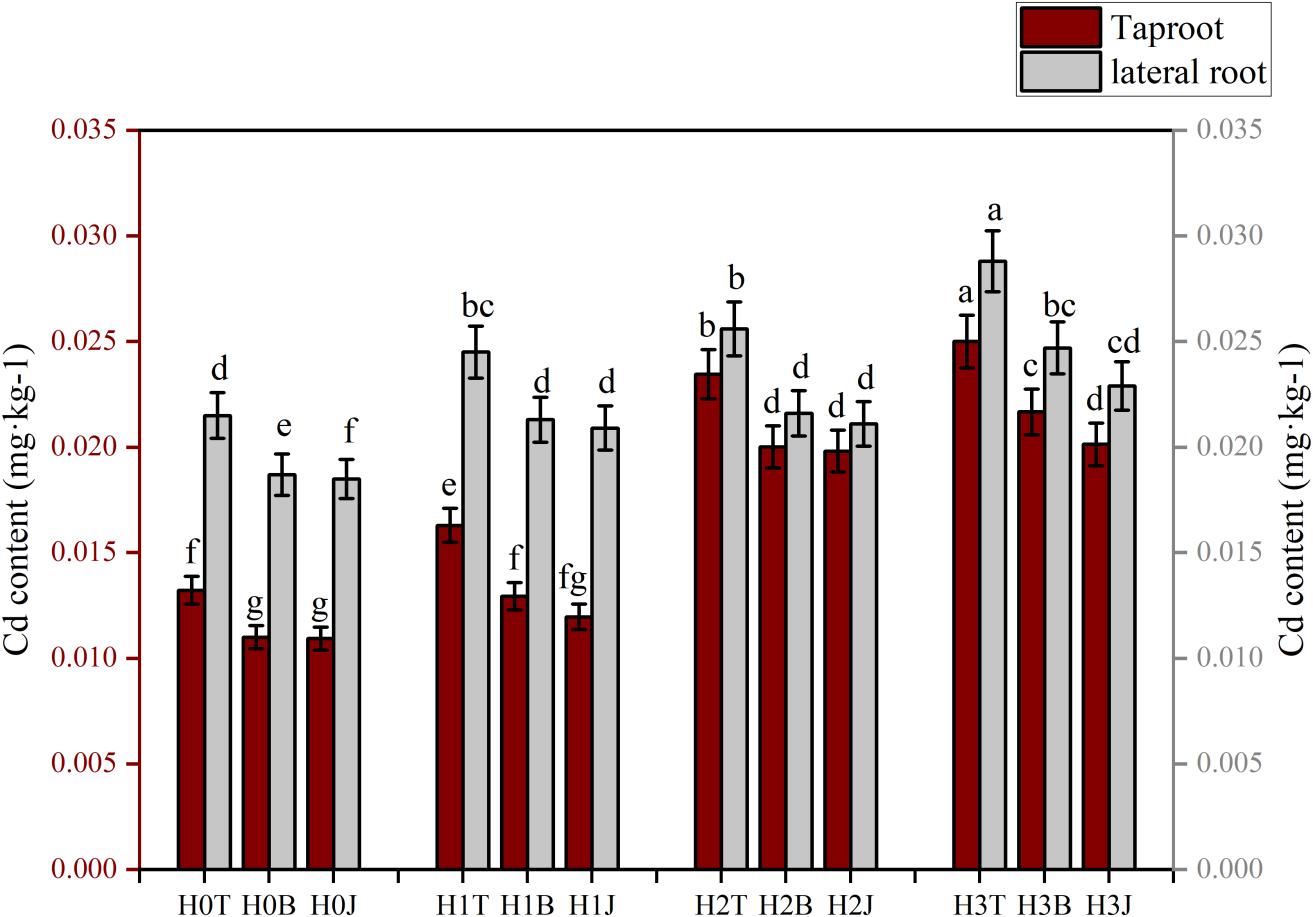
Effect of biochar and biofertilizer applications on cotton root Cd content. T, no modifiers; B, 3% biochar was applied; J, 1.5 % biofertilizer was applied; H0, no Cd; H1, 1 mg·kg-1 of Cd was applied; H2, 2 mg·kg-1 of Cd was applied; H3, 4 mg·kg-1 of Cd was applied. Values are means ± SE (n=5). Different lowercase letters indicate significant differences at P < 0.05 (the Duncan test).

As shown in **Figure 4**, there were statistically significant differences between the treatments, and all samples were within the 95% confidence interval. **Figure 4A** shows the cation grouping information in the measured samples, and **Figure 4B** shows anions. The samples in the H3B and H3J treatments showed obvious differences.

### Metabolic pathway and expression pattern analysis of DEMs

**Figure 5:**
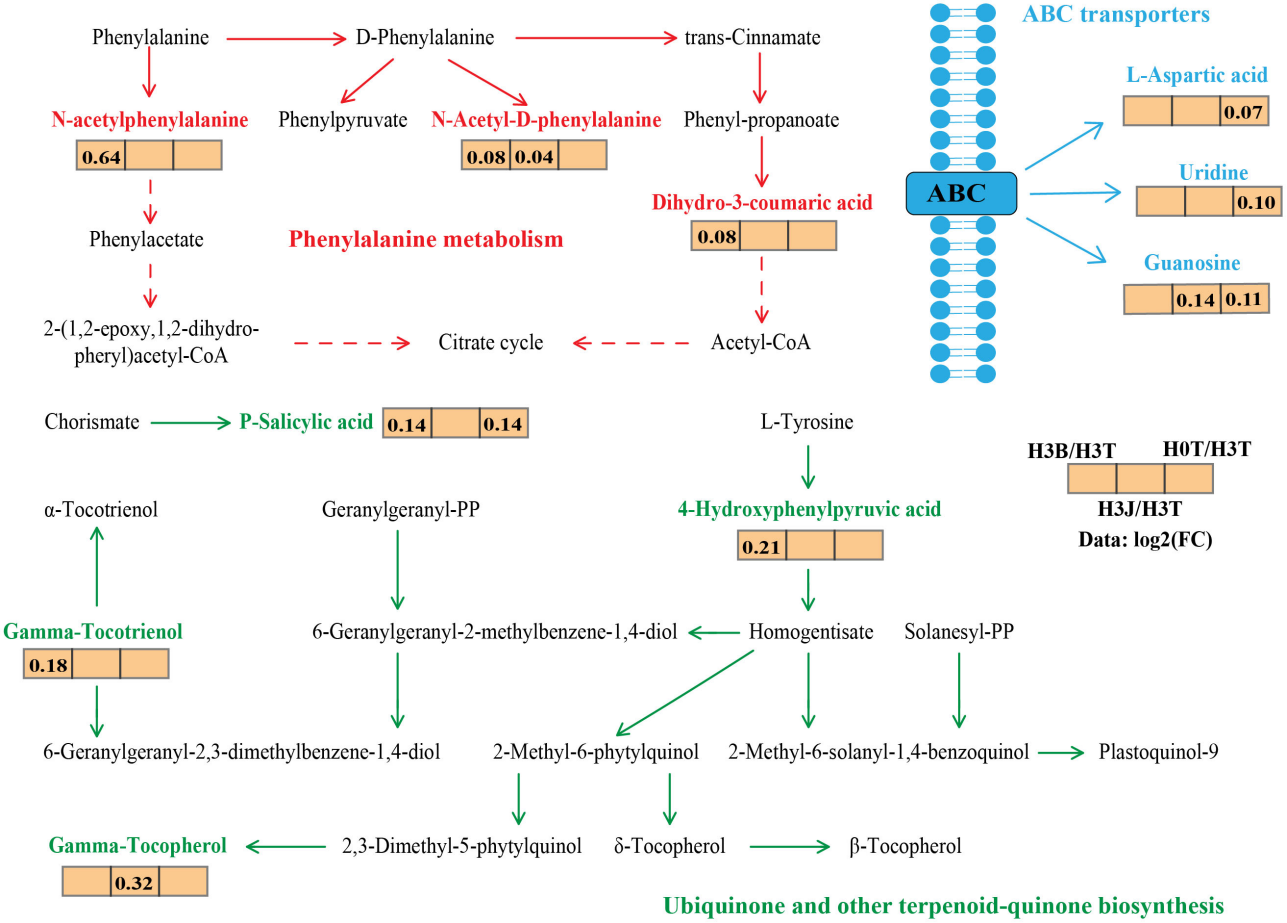
PLS-DA analysis of untargeted metabolomics data of cotton lateral root in the Cd, biochar, and biofertilizer treatments (left, cation) (right, anion). Ellipses represent the 95% confidence interval.

The main DEMs in cotton lateral root in the treatments were selected through pairwise comparison (**Figure S1**). The network of main DEMs (**Figure 5**) showed that in ABC transporters, L-aspartic acid, Uridine, and Guanosine were the main DEMs, and their relative abundance were up-regulated after Cd addition. In Phenylalaninc metabolism, the relative abundance of the main DEMs N-acetylphenylalanine, N-acetyl-d-phenylalanine, and Dihydro-3-coumaric acid in the H3B treatment were up-regulated compared with those in the H0T treatment. In Ubiquinone and other terpenoid quinone biosynthesis, the relative abundance of 4-hydroxyphenylpyruvic acid, P-salicylie acid, and Gamma tocotrienol in the H3B treatment and that of Gamma tocotrienolu in the H3J treatment were up-regulated compared with those in the H0T treatment. The Cd addition reduced the relative abundance of Gossypol (**Figure S2**).

### Interaction network between the primary metabolism and environment factors

**Figure 6:**
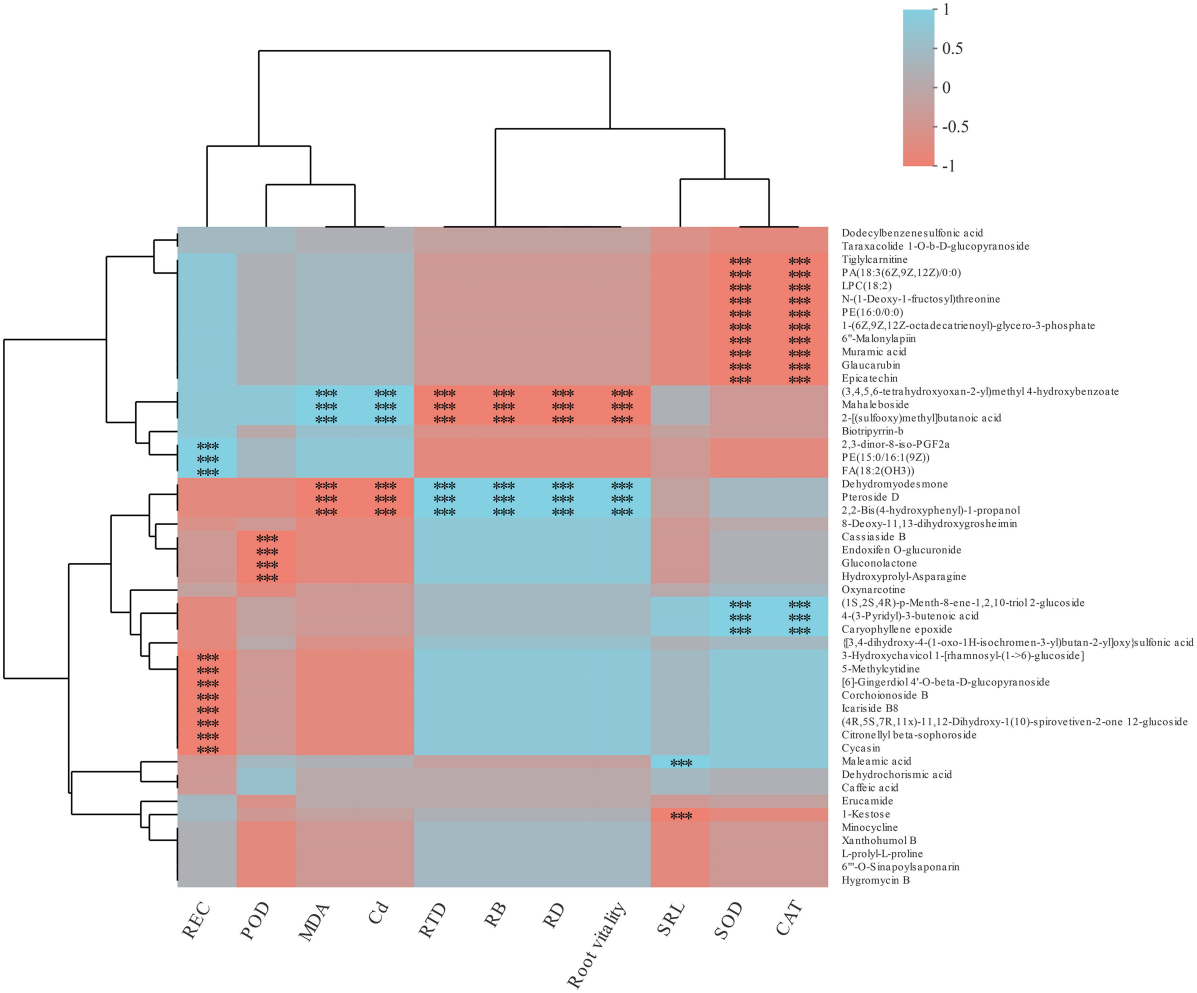
Primary metabolic flux map labeled with the differentially expressed metabolites of cotton lateral root. The data represents the ratio of the average expression of metabolites in the two groups of samples (Log2FC), with positive value indicating up-regulation and negative value indicating down-regulation. Solid arrows indicate direct action and dashed arrows indicate indirect action. Red represents Phenylalaninc metabolism; Green represents Ubiquinone and other terpenoid-quinone biosynthesis; Blue represents ABC transporters.

The main DEMs in cotton lateral root were screened (e.g: PE(15:0/16:1(9z), dodecylbenzenesulfonic acid, and some organic acids) (**Figure S2**) to construct the heatmap of cotton root morphology, physiological indexes, and metabolites (**Figure 6**). It was found that root Cd content, MDA content, RTD, RB, RD, and root activity were negatively correlated with the relative abundance of (3.4.5.6-ethoxyoxan-2-y)methyl 4-hydroxybenzoate, Mahaleboside, and 2-[(sulfoxy)methyl]butanoic acid (*p<* 0.01), and positively correlated with the relative abundance of Dehydromodesmone, Pteroside D, and 2,2-Bis-(4-hydroxyphenyl)-1-propanol (*p<* 0.01).

SOD and CAT activities of cotton lateral root were negatively correlated with the relative abundance of Tiglylcarmitine, PA(18:3(6Z,9Z,12Z)0:0), LPC(18:2), N-(1-deoxy-1-fluorosyl)threonine, PE(16:0/0:0), 1-(6Z.9Z.12Z-octadecarieny)-glycoro-3-phosphate, 6”-malonylapin, Muramic acid, Glaucarubin, and Epicatechin (*p*< 0.01), and positively correlated with (1S.2S.4R)-p-menth-8-ene-1.2.10-riol 2-glucoside, 4-(3-pyridyl)-3-butenolc acid, and Caryophyllene epoxy (*p*< 0.01). REC was negatively correlated with 3-Hydroxychavicol 1-thamnosyl-(1->6)-glucoside, 5-methylcytidine, 6-gingerdiol 4-0-beta-d-glucopyranoside, Corchoionoside B, lcariside B8, (4R,5S,7R.11)-11,12-Dihydroxy-1(10)-spirovetiven-2-one 12-glucoside, Citronellyl beta-sophoroside, and Cycasin (*p*< 0.01), and positively correlated with 2,3-dinor-8-IS0-PGF2a, PE(15:0/16:1(9Z), and FA(18:2(OH3). POD activity was negatively correlated with Cassiaside B, Endoiten o-glucuronide, Gluconolactone, Hydroxyprolyl-asparagine, and Oxynarcotine (*p*< 0.01).

**Figure 7:**
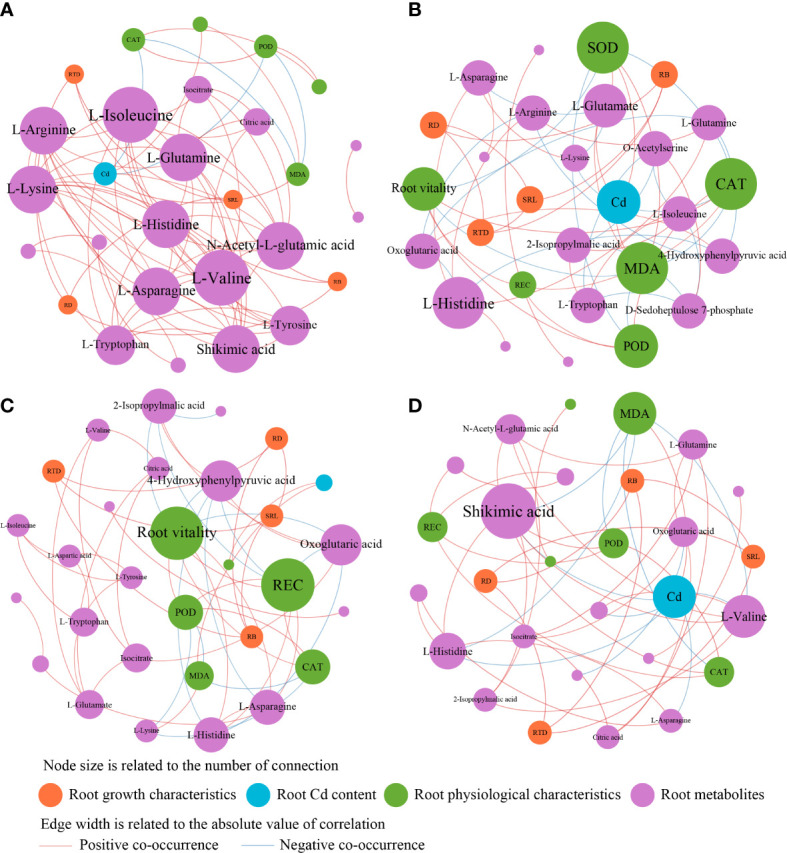
Heatmap of the correlation between metabolites and environmental factors in each treatment. REC, electrolyte leakage rate; MDA, malondialdehyde; POD, peroxidase; SOD, superoxide dismutasee; CAT, catalas; RTD, root tissue mass density; RB, root biomass; RD, specific root length density; SRL, specific root length; Cd, root Cd content.

The different responses of cotton lateral root metabolites to the treatments led to differences in the physiological and growth characteristics of cotton lateral roots and the interactions between metabolites. Therefore, the interactions of root growth characteristics, Cd content, physiological characteristics, and metabolites in different treatments were analyzed by using the co-occurrence network (**Figure 7**). In H0T treatment, the main metabolites were L-isolecine, L-valine, L-argininc, Lasparagine, and Shikimic acid (**Figure 7A**). In the H3T treatment, exogenous Cd addition increased Cd content in cotton roots and decreased SOD and CAT activities and root vitality. Besides, it also increased the relative abundance of key metabolites including L-glutamate, L-histidine, L-arginine, and 4,hydroxyphenylpyruvic acid (**Figure 7B**). In the H3B treatment, B application increased cotton root vitality, REC, and the relative abundance of 4-Hydrophenylpyruvic acid and Oxoglutaric acid (**Figure 7C**). In the H3J treatment, J application reduced cotton root Cd content, MDA content, and the relative abundance of Shikimic acid (**Figure 7D**).

## Discussion

Taproot and lateral roots are the main parts enriched by Cd in cotton plants (Nan et al., 2020). The development of lateral roots has a great influence on plants. Massive lateral roots can increase the plants’ uptake of nutrients, water, and metal ions (Wang et al., 2020). In this study, exogenous Cd addition reduced cotton RB, RTD, and root vitality, but had no obvious effect on RD and SRL (**Table 3**). This is different from the research results of Ng et al. (2019). This difference is mainly caused by the differences in the root growth characteristics of different plants and the sensitivity to different metal elements (Ng et al., 2020). In addition, in this study, the application of 4 mg kg^-1^ Cd decreased the vitality of cotton taproot and lateral root by 45.45% and 56.73%, respectively compared with the control (**Figure 1**). This indicates that exogenous Cd addition could inhibit the growth and development of cotton root system (Lambrechts et al., 2014).

The application of B and J increased RB, SRL, RD, and root vitality, and reduced the content of bioavailable Cd in soil, thus inhibiting the absorption of Cd by plants and alleviating Cd stress (Liu et al., 2008; Jha et al., 2020). Shah and Nahakpam (2012) showed that the POD and CAT activities of rice root increased under Cd stress. This is contrary to the results of the present study (**Figure 3**). It may be caused by differences in the scavenging of reactive oxygen species, the reduction of lipid membrane peroxidation, and the adaptability to adverse environments in different crops (Sun et al., 2017; Elsayed et al., 2020). Bashir et al. (2020) showed that the application of B and J increased the activities of POD and SOD in wheat roots in Zn-contaminated soil. This is consistent with the results of this study. The application of B and J increased the activities of POD and CAT in cotton root (**Figure 2**), and reduced MDA content and REC (**Figure 3**). This may be due to that B and J could reduce the accumulation of Cd in cotton root by adsorbing and fixing Cd (Ogunkunle et al., 2018).

**Figure 8:**
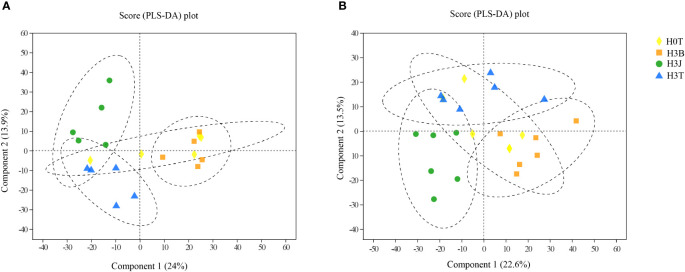
Co-occurrence analysis of root growth characteristics, root Cd content, root physiological characteristics, and root metabolites in response to different treatments. (a) H0T treatment, (b) H3T treatment, (c) H3B treatment, (d) H3J treatment.

In this study, after 1, 2, 4 mg kg^-1^ of Cd were applied, the Cd content in cotton taproot and lateral root increased by 89.11% and 33.95% on average (**Figure 8**) compared with those in the control, and the Cd content of cotton lateral root was higher than that of taproot. This may be due to that the increase of cotton lateral roots not only increases the absorption of water and nutrients, but also increases the absorption of metal ions (Wang et al., 2020). Besides, it was also found that after the application of B and J, the Cd content of cotton roots decreased by 13.31%-26.63% and 13.02%-20.49%, respectively compared with that of the control. This may be due to that (1) B and J are rich in carboxyl groups and minerals, which can improve soil fertility and structure and fix toxic elements, thereby reducing the uptake of toxic elements by plants (Elsayed et al., 2020; Jha et al., 2020); Besides, B and J can also promote plant growth and development. (2) The application of B and J can increase soil microbial diversity to produce more organic acids and enhance the adsorption and fixation of toxic elements by soil microorganisms (Zhang et al., 2020). (3) The application of B and J can stimulate plant roots to produce more exudates, increasing the release of toxic elements (Cheng et al., 2018; Vinci et al., 2018).

Under heavy metal stresses, amino acids and their derivatives secreted by plants play important roles in resisting the toxicity of metal ions (Anjum et al., 2015; Olmo et al., 2016). Studies have shown that increases in the contents of alanine, proline, serine and putrescine in plant root exudates can alleviate the Cd stress on plants (Wang et al., 2015). In this study, when 4 mg kg^-1^ of Cd was applied, the relative abundance of DEMs including L-aspartic acid, Uridine, and Guanosine were up-regulated (**Figure 6**, **Figure S3**). Wang et al. (2015) and Yang and Shen (2020) showed that when the Cd content was 1 mg kg^-1^, the secretion of amino acids and organic acids by the roots of *Typha latifolia* L. significantly increased, but the content of organic acids decreased significantly with the increase of Cd concentration. However, in this study, the relative abundance of L-aspartic acid increased after exogenous Cd addition. This may be related to the amount of exogenous Cd. Different Cd contents have different toxicity to plants and microorganisms, resulting in different antitoxicity of plants. In addition, the species difference between *Typha latifolia* L. and cotton and different planting environments may also lead to difference in plant root exudates (Yang and Shen, 2020).

Studies have also shown that differences in heavy metals and cultivation conditions have significant impacts on the composition of plant metabolites (Fu et al., 2017). That is, plants can regulate their metabolic intensity to improve their adaptability to the polluted environment (Yang and Shen, 2020). In this study, cotton root exudates were rich in organic acids and amino acids (**Figures S2-4**). This indicates that under Cd stress, cotton mainly secretes organic acids and amino acids to resist Cd stress, which is consistent with the results of Fan et al. (2016) and Arif et al. (2019). In this study, there was a negative correlation between root Cd content and Pteroside D abundance (*p<* 0.01) (**Figure 6**). Pteroside D is a kind of carbohydrates. Heavy metals have a great impact on the synthesis of carbohydrates in plants. Excessive accumulation of heavy metals may inhibit the synthesis of carbohydrates by breaking the photosynthetic electron transport chain and producing ROS, thus affecting the photosynthesis and nutrient absorption of plants (Khan et al., 2015). It was also found that root Cd content was closely related to amino acids (**Figures 7A, D**). This indicates that amino acids play important roles in the resistance to Cd stress. After B and J were applied, the relative abundance of N-acetylphenylalanine, N-acetyl-D-phenylalanine, and Gamma-tocotrienol increased (**Figure 5**), and the activities of root antioxidant enzymes were closely related to root exudates (**Figures 7B, C**). This indicates that phenolic substances and phenylalanine secreted by cotton roots play important roles in antioxidation and the resistance to Cd stress (Kováčik and Bačkor, 2007). However, An et al. (2022) found that after applying polymer modifier to Cd-contaminated soil, cotton leaves mainly secreted jasmonic acid, stearic acid, and other organic acids to resist Cd stress, and no amino acids were found. This difference may be caused by the type or amount of modifiers applied.

Epicatechin and coumaric acid are closely related to the biosynthesis pathway of flavonoids and phenolic acids, which can enhance the free radical scavenging ability and the oxidative stress response of plants (Banik and Bhattacharjee, 2020). In this study, SOD and CAT activities of cotton root were negatively correlated with the relative abundance of Muramic acid, Glaucarubin, and Epicatechin (*p*< 0.01) (**Figure 6**), and positively correlated with the relative abundance of L-glutamate, L-asparagine, and 4-hydroxyphenylpyruvic acid (**Figures 7C, D**). This indicates that the application of B and J could improve the resistance to Cd stress through improving the antioxidant defense system and increasing the secretion of amino acids by cotton root. In this study, the relative abundance of Mahaleboside and 2-[(sulfooxy)methyl]butanoic acid were positively correlated with RD, root biomass, and RTD (*p*< 0.01) (**Figure 6**). Mahaleboside is a kind of phylpropanoids and polyketides, which plays an important role in the metabolic pathway of plant defense system and nutritional synthesis. It can enhance cotton resistance to abiotic stress and promote cotton growth and development (Wu et al., 2016; Li et al., 2018). Cotton root vitality and root growth indicators were positively correlated with Pteroside D (*p*< 0.01) (**Figure 6**). Pteroside D is a kind of carbohydrates. Root growth and development consume a large amount of carbohydrates produced by energy metabolism (Singh et al., 2015; Sun et al., 2017). Therefore, B and J application could regulate the growth indicators (RTD, SRL, and RD) and metabolites (organic acids and amino acid compounds) of Cd-stressed cotton root to reduce the absorption of Cd by cotton taproot and lateral roots (**Figures 7B, C**), thus reducing the Cd stress on cotton growth.

## Conclusion

Exogenous Cd application inhibited the growth and development of cotton roots (RB, RTD, and root vitality), and reduced the antioxidant enzyme activities. However, applications of cotton straw-derived biochar and compound *Bacillus* biofertilizer promoted root growth and root vitality, and increased the SOD and CAT activities of Cd-stressed cotton root. Besides, cotton straw-derived biochar and compound *Bacillus* biofertilizer mainly regulate the relative abundance of cotton root metabolites including phenolic substances and amino acids to reduce the absorption of Cd by cotton roots, thus improving cotton resistance to Cd stress.

## Data availability statement

The original contributions presented in the study are included in the article/**Supplementary Material**, further inquiries can be directed to the corresponding author.

## Author contributions

YZ and JC conceived and designed the experiments. HW and JS conducted laboratory analyses. YZ and MZ analyzed the data and wrote results. YZ, XL, and TL wrote the manuscript (Introduction and Discussion). All authors provided editorial advice and revised manuscript.

## Funding

This work was supported by the National Natural Science Foundation of China (Grant No. 42161042), the Corps Science and Technology Project (Grant No.2020AB018), and the Shihezi University Project (Grant No. RCZX201425 and RCZK20208).

## Conflict of interest

The authors declare that the research was conducted in the absence of any commercial or financial relationships that could be construed as a potential conflict of interest.

## Publisher’s note

All claims expressed in this article are solely those of the authors and do not necessarily represent those of their affiliated organizations, or those of the publisher, the editors and the reviewers. Any product that may be evaluated in this article, or claim that may be made by its manufacturer, is not guaranteed or endorsed by the publisher.

## Appendix A. Supplementary material

All data generated or analyzed during this study are available from the corresponding author on reasonable request.
