# Supplementary material for: Cotton straw biochar and compound Bacillus biofertilizer reduce Cd stress on cotton root growth by regulating root exudates and antioxidant enzymes system
Source: Front Plant Sci. 2022 Nov 15;13:1051935. doi: 10.3389/fpls.2022.1051935 (PMC9705756; doi:10.3389/fpls.2022.1051935)
Supplement: Supplementary file 1 [file DataSheet_1.docx]

**Supplementary information:**

**Complementary information about cotton root metabonomics.**


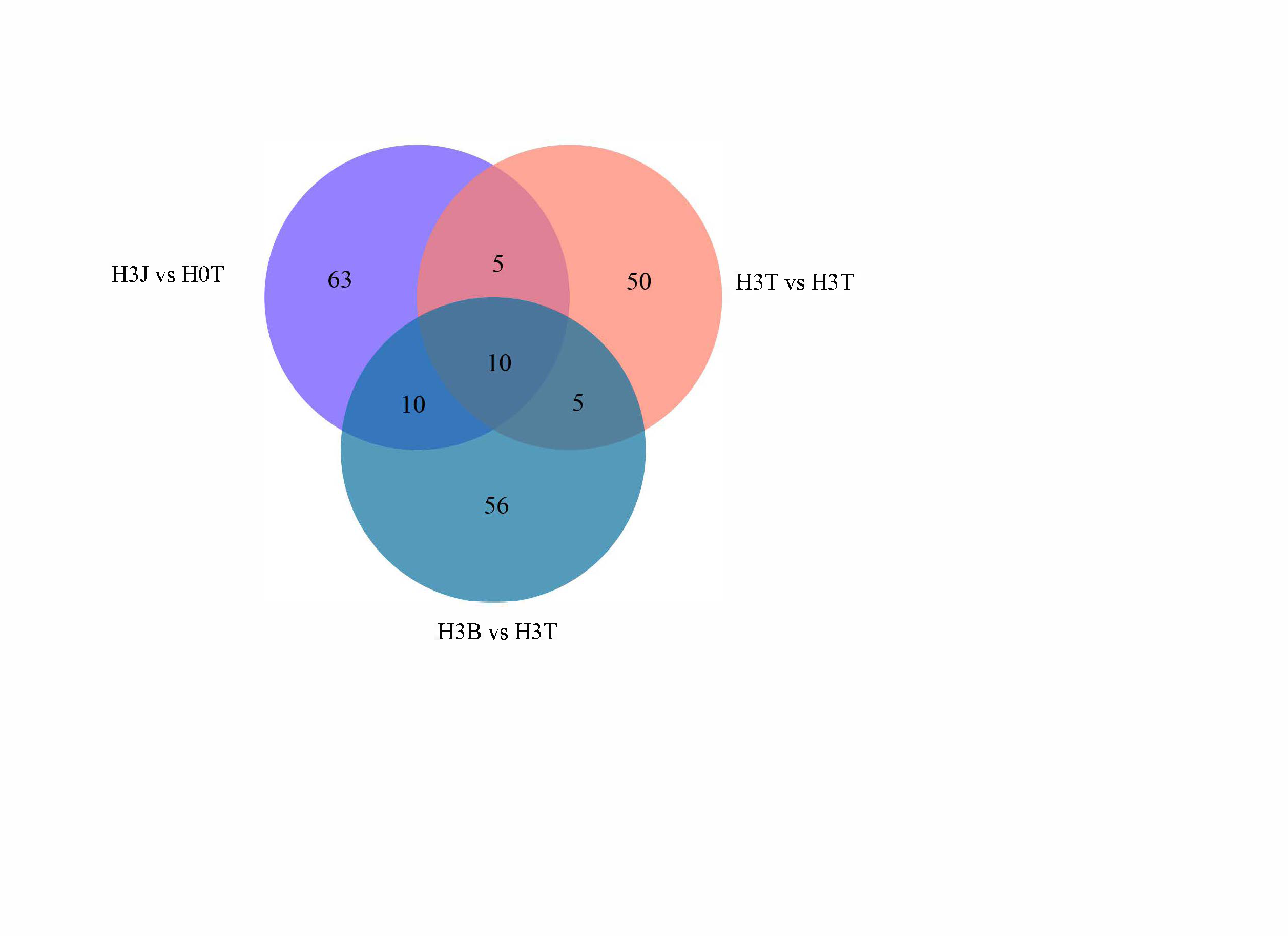


**Fig. S1**. Comparison of cotton metabolomics data for different treatments. Venn diagrams displaying (comparatively) the differentially expressed metabolites. T, no modifiers; B, 3% biochar was applied; J, 1.5 % biofertilizer was applied; H0, no Cd; H1, 1 mg·kg^-1^ of Cd was applied; H2, 2 mg·kg^-1^ of Cd was applied; H3, 4 mg·kg^-1^ of Cd was applied. The same below. All differentially expressed metabolites are clustered into three comparison groups represented by three circles. The sum of all the figures in one circle represents the number of differentially expressed metabolites in one comparison group (e.g., H3J vs. H3T, H3B vs. H3T, H3T vs. H0T). The overlapping parts of different circles represent the number of differentially expressed metabolites shared between these comparison groups. The single-layer part represents the number of metabolites distinctly found in a certain comparison group.


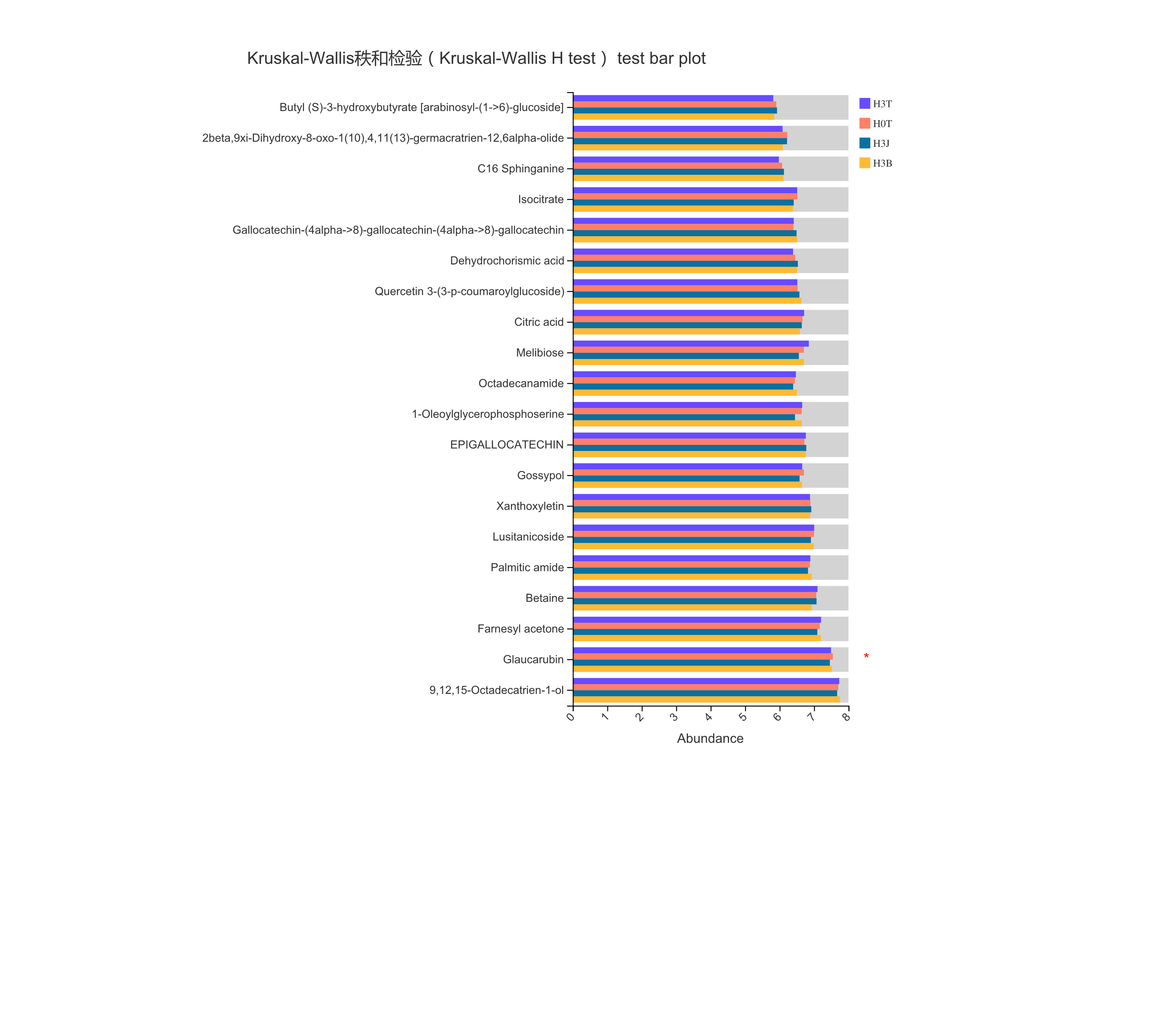


**Fig. S2.** Multi-group comparison of differentially expressed metabolites in cotton roots in different treatments (Kruskal-Wallis H test). The ordinate represents the name of metabolites, the abscissa represents the average relative abundance of metabolites in different groups, and different colored columns represent different groups. The far right shows the significance of the differences, * *0.01 ≤ p ≤ 0.05*, ** *0.001 ≤ p ≤ 0.01*, *** *p ≤ 0.001*.


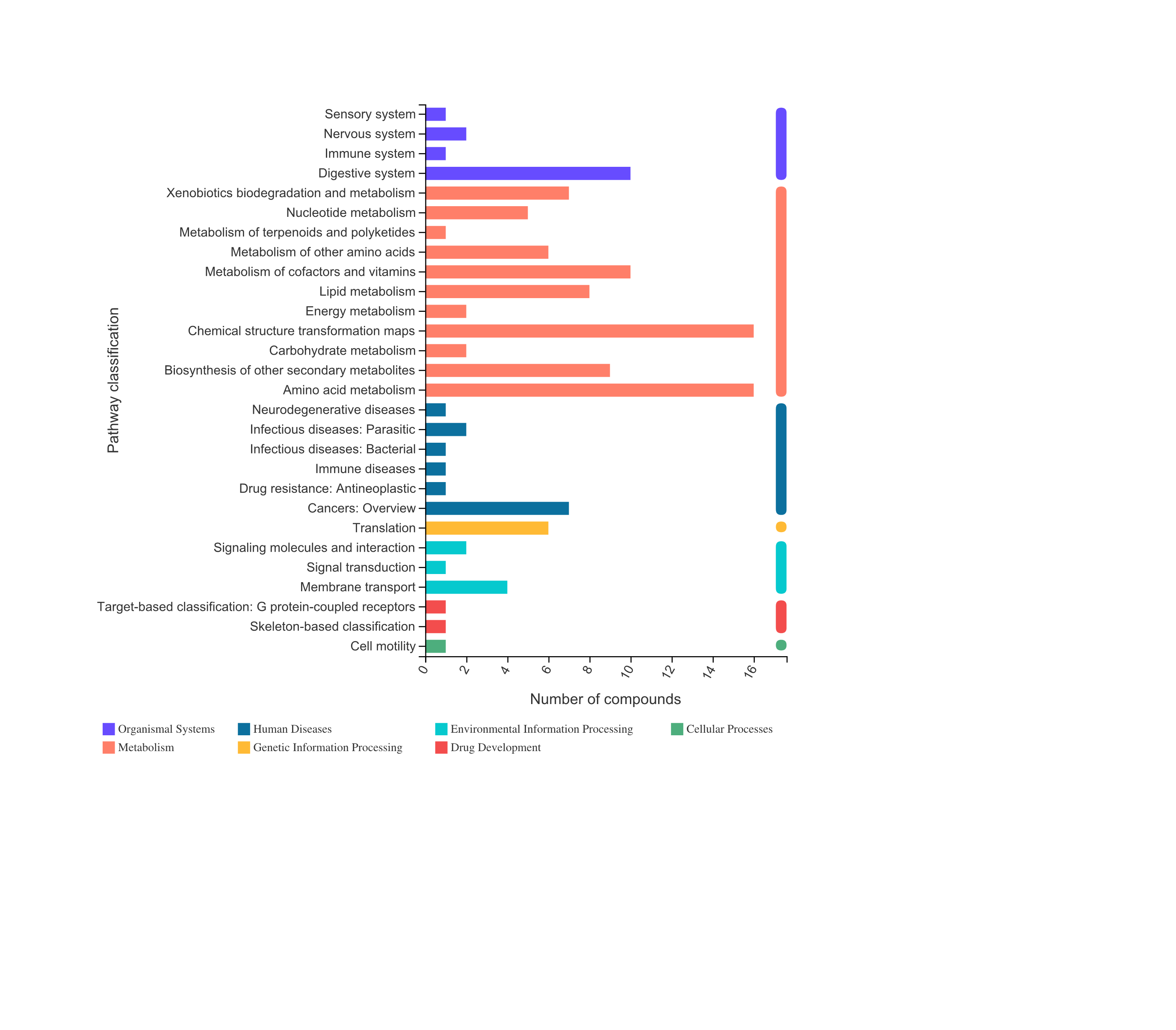


**Fig. S3.** KEGG pathway classification: metabolites detected and annotated (POS model). The abscissa represents level-2 terms of the KEGG pathway and the ordinate represents the number of metabolites identified.


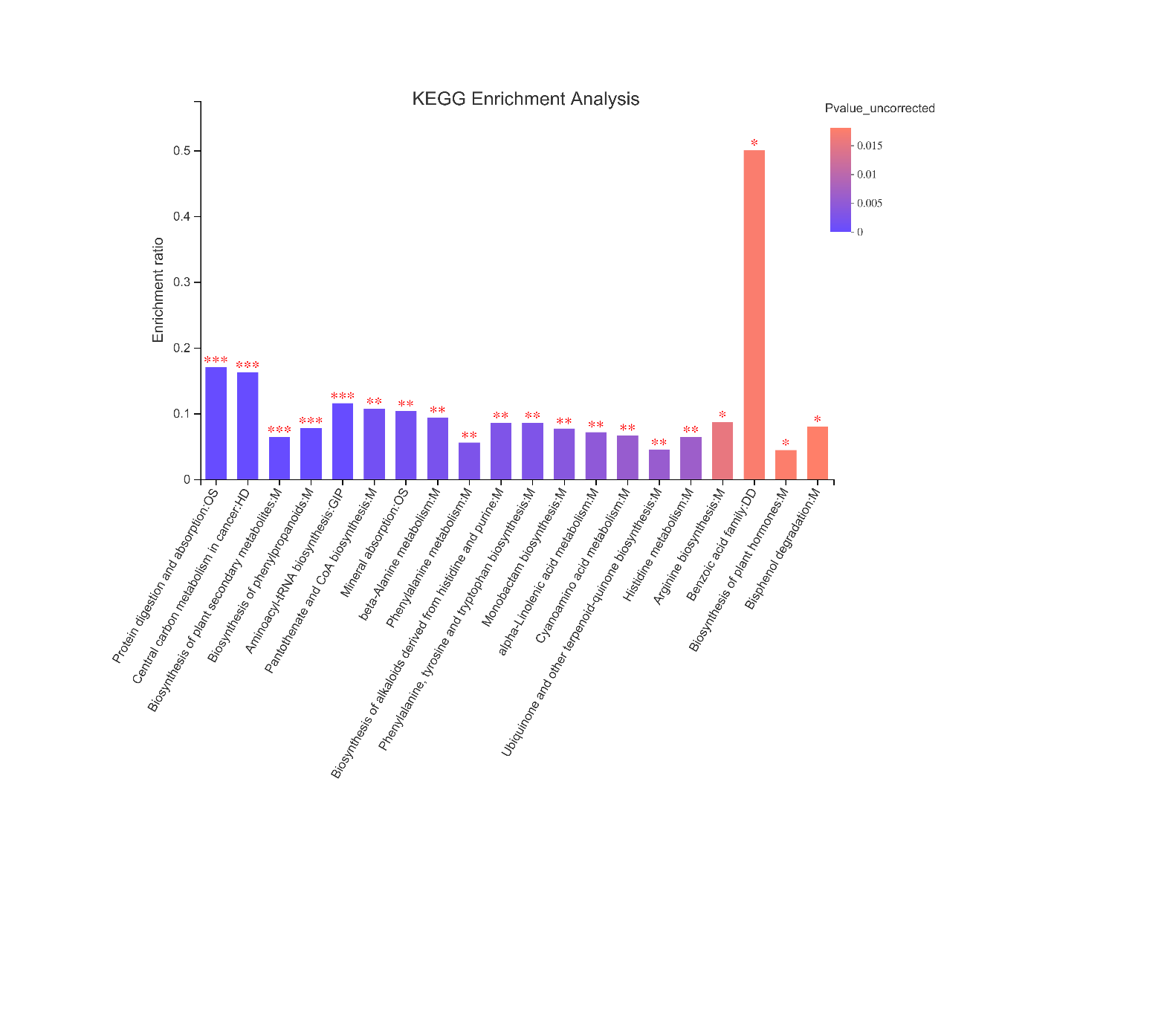


**Fig. S4.** Metabolic pathway enrichment analysis. Overview of metabolites that were enriched in the biochar group compared to the biofertilizer group. DD, HD, M, and OS are the class names of the metabolic pathways in the KEGG annotation. DD: Drug Development; HD: Human Diseases; M: Metabolism; OS: Organismal Systems. The abscissa represents pathway name, and the ordinate represents enrichment rate, representing the ratio between the number of metabolites enriched in this pathway (Metabolite number) and the number of background number of metabolites annotated into this pathway. The greater the ratio, the greater the degree of enrichment. Colour gradient of the column represents the significance of enrichment. The darker the default color is, the more significant the enrichment of the KEGG term, *p* < 0.001, ***, *p* < 0.01 **, *p* < 0.05 *.
